# Complement Deposition Predicts Worsening Kidney Function and Underlines the Clinical Significance of the 2010 Renal Pathology Society Classification of Diabetic Nephropathy

**DOI:** 10.3389/fimmu.2022.868127

**Published:** 2022-05-27

**Authors:** Shimin Jiang, Dingxin Di, Yuanyuan Jiao, Guming Zou, Hongmei Gao, Wenge Li

**Affiliations:** ^1^ Department of Nephrology, China-Japan Friendship Hospital, Beijing, China; ^2^ Graduate School of Peking Union Medical College, Peking Union Medical College and Chinese Academy of Medical Sciences, Beijing, China

**Keywords:** complement system, C3, RPS classification, diabetic nephropathy, prognosis

## Abstract

**Objectives:**

Converging evidence points towards a role of the complement system in the pathogenesis of diabetic nephropathy (DN). The classification system of diabetic kidney lesions devised by the Renal Pathology Society (RPS) in 2010 are based on the pathogenic process of DN. Therefore, we investigated the correlation between glomerular C3 deposits and RPS DN classification and the combined deleterious effects thereof on kidney function.

**Methods:**

The study analyzed data from 217 diabetic patients who underwent renal biopsy between 2010 and 2021 and were found to have DN as the only glomerular disease. C3 deposition was considered positive if the glomerular C3 immunofluorescence intensity was at the trace or ≥1+ level. We divided DN into five glomerular lesion classes and separately evaluated the degree of interstitial and vascular involvement. The primary outcome was the composite of a ≥50% decline from the initial estimated glomerular filtration rate, end-stage renal disease, and death.

**Results:**

None of the patients were classified into class I, and few were classified into classes IIa (7.8%) and IV (9.2%). Most patients were classified as IIb (30.9%) and III (52.1%). C3 deposition was detected in 53.9% of patients. Multivariate logistic regression analysis showed that DN class was significantly correlated with C3 deposits [odds ratio, 1.59; 95% confidence interval (CI), 1.08–2.36; *p* = 0.02). During a median follow-up of 22 months, 123 (56.7%) patients reached the composite outcome. The endpoints occurred more frequently in patients with C3 deposition (69.2 vs. 42%) compared with those without C3 deposition. Patients with C3 deposition in either class IIb [hazards ratio (HR), 3.9 (95% CI, 1.14–13.17) vs. 2.46 (95% CI, 0.68–8.89)] or III [HR, 4.98 (95% CI, 1.53–16.23) vs. 2.63 (95% CI, 0.77–9.0)] had a higher risk of adverse kidney outcomes than those without C3 deposition. The prognostic accuracy of the combination of DN class and C3 deposits at 1 and 3 years was higher than that for DN class only.

**Conclusions:**

Complement deposition together with DN class predicts more rapid deterioration of kidney function in DN, which underlines the clinical significance of the DN phenotype according to the RPS classification.

## Introduction

In the past two decades, the estimated prevalence of diabetes in adults has increased 3-fold, from 151 million (4.6% of the global population in 2000) to 537 million (10.5%) today (1). The increasing prevalence of diabetic nephropathy (DN) has paralleled the staggering worldwide rise in the prevalence of diabetes (2). DN remains the most common cause of end-stage renal disease (ESRD). Therefore, DN is a vital clinical issue, and an in-depth understanding of its underlying mechanisms will aid in the development of new therapeutic strategies.

The pathogenesis of DN is complex and involves multiple pathways previously classified as metabolic, hemodynamic, intracellular, and growth factor/cytokine-related (3). Increasing evidence also supports a role of the innate immune system in inflammatory processes related to diabetes and DN (4). As a moderator of the innate immune system, the complement system has a key role in lysing, damaging, and activating target cells (5).

Complement deposition has been observed in the kidneys of DN patients and may cause kidney injury (6–8). There are two main putative mechanisms for the involvement of the complement system in the development of DN (4): activation of the lectin pathway in response to glycated proteins due to overexposure to glucose and glycation of complement-regulated proteins induced by hyperglycemia (which leads to the dysfunction of their regulatory capacity). Recent evidence showed that the classic complement pathway may also be involved in kidney damage in DN patients (6, 9). The three complement pathways (classic, alternative, and lectin) lead to the formation of complement C3, which generates the membrane attack complex that triggers sequential downstream cascades.

In 2010, the Renal Pathology Society (RPS) devised a DN classification scheme to standardize the identification and scoring of kidney lesions (10). Lesions are classified based on the glomerular compartment and a single score pertaining to the tubulointerstitial and vascular compartments; this system is easy to use in clinical practice. The morphological lesions are a consequence of the pathogenic process of DN. Given the role of complement activation in DN, it would be of interest to explore whether the complement system can affect the pathologic phenotype of DN. Thus, we investigated the association of glomerular C3 deposits with the RPS DN classification and the combined deleterious effects thereof on kidney function.

## Patients and Methods

### Study Population

**Figure 1:**
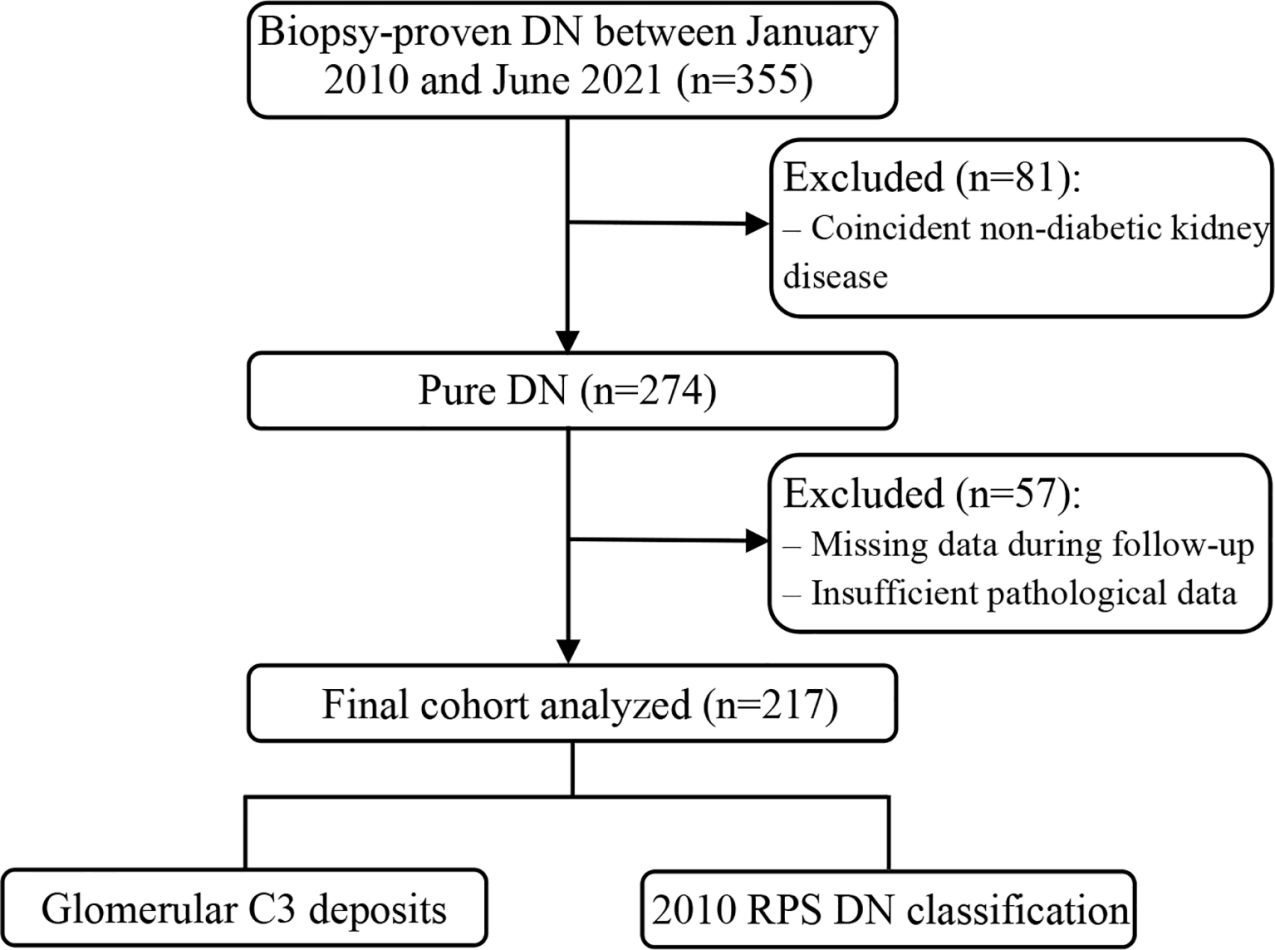
Flow chart of the study participants. DN, diabetic nephropathy; RPS, Renal Pathology Society.

This observational study included 355 patients with biopsy-proven DN diagnosed at our center between January 2010 and June 2021. Diabetes was classified as type 1 or 2 and diagnosed based on the fasting blood glucose level, 2-h postprandial plasma glucose level during a 75-g oral glucose tolerance test, or glycated hemoglobin A1c (HbA1c) level, according to the 2022 American Diabetes Association criteria (11). DN was diagnosed on the basis of suggestive clinical and pathological features, including increased mesangial matrix (nodular glomerulosclerosis) and diffuse glomerular basement membrane thickening on renal histology (12). Patients with a history of kidney transplantation, superimposed glomerular disease, lacking follow-up data, or inadequate biopsy core tissue (<5 glomeruli) were excluded. A flow diagram of the selection of study participants is provided in **Figure 1**.

### Clinical and Biochemical Data

Clinical and laboratory data were extracted from electronic medical records at the time of renal biopsy and included age, sex, body mass index, blood pressure, diabetes duration, presence of diabetic retinopathy and/or neuropathy, presence of microscopic hematuria, urinary protein excretion (UPE) over 24 h, and levels of HbA1c, hemoglobin, serum albumin, creatinine, uric acid, blood urea nitrogen, fasting blood glucose, total cholesterol, triglycerides, low-density lipoprotein cholesterol, high-density lipoprotein cholesterol, and serum C3, C4, and C1q. In addition, we recorded the use of angiotensin-converting enzyme inhibitor (ACEI)/angiotensin II type 1 receptor blocker (ARB) therapy and the history of cardiovascular events (CVEs). Mean arterial pressure was calculated as diastolic pressure + 1/3 (systolic pressure – diastolic pressure).

Microscopic hematuria was defined as the excretion of >3 erythrocytes per high-power field in urine sediments from at least two consecutive samples. CVEs were defined as a history of definitive diagnosis of cardiovascular diseases, including myocardial infarction, angina, acute coronary syndrome, revascularization events (coronary artery surgery and percutaneous transluminal coronary angioplasty), significant coronary stenosis, and stroke.

### Histological Data and Pathological Classification

The renal biopsy specimens were processed at our center and evaluated by light, immunofluorescence, and transmission electron microscopy. Renal biopsy specimens were reviewed by investigators blinded to the clinical outcomes. DN was classified and graded histologically according to the 2010 RPS DN classification (10). The glomerular compartment was classified as class I–IV. Light microscopy changes in the glomerular basement membrane and epithelial foot process effacement on electron microscopy had no influence on the classification. Class I included isolated glomerular basement membrane thickening and only mild, non-specific light microscopy changes that did not fulfil the criteria of classes II–IV. Class II included mild (IIa) or severe (IIb) mesangial expansion that did not fulfil the criteria for class III or IV. Class III included nodular sclerosis, defined as the presence of at least one convincing Kimmelstiel–Wilson lesion and ≤50% global glomerulosclerosis. Class IV included advanced DN, defined as >50% global glomerulosclerosis. Interstitial fibrosis and tubular atrophy (IFTA) were graded together as a percentage of the total area of interstitium and tubule involvement as follows: 0, absent; 1, <25%; 2, 25–50%; or 3, > 50%. Arteriolar hyalinosis was scored as 0 if no arteriolar hyalinosis was present, 1 if one arteriole with hyalinosis was present, and 2 if more than one arteriole with hyalinosis was observed. Arteriosclerosis was graded in the most severely affected artery as follows: 0, no intimal thickening; 1, intimal thickening less than the thickness of the media; and 2, intimal thickening greater than the thickness of the media. The scores were assigned by the same pathologist.

The intensity of direct immunofluorescence staining of C3 deposits in kidney tissue was graded using a semiquantitative method on a scale of 0–4+ (–, no fluorescence at either low or high magnification; ±/trace, no fluorescence at low magnification but somewhat visible at high magnification; +, somewhat visible at low magnification but clearly visible at high magnification; ++, clearly visible at either low or high magnification; +++, clearly visible at low magnification but dazzling at high magnification; and ++++, dazzling at low magnification and even more dazzling at high magnification) (13). In this study, the C3 deposits were graded on the basis of the presence or absence of glomerular deposits, including deposition in glomeruli only and co-deposition in glomerular and tubulointerstitial compartments.

### Follow-up and Endpoints

A 5-year patient follow-up was planned. The primary endpoint was a composite of ESRD onset, ≥50% decline in baseline estimated glomerular filtration rate (eGFR), and death during follow-up. Survival time was defined as the interval from the time of kidney biopsy to one of the three endpoints: composite outcome, loss to follow-up, or the study end date. ESRD was defined as the need for long-term dialysis or kidney transplant. The eGFR was calculated using the creatinine-based Chronic Kidney Disease Epidemiology Collaboration equation (14).

### Statistical Analysis

Continuous variables are expressed as mean and standard deviation (SD) for data with a normal distribution and as median with interquartile range (IQR) for data with a skewed distribution. Categorical variables are presented as count (*n*) and percentage (%). Normally distributed variables were compared using one-way analysis of variance (ANOVA), while skewed variables were analyzed by Kruskal–Wallis or Mann–Whitney *U*-test as appropriate. Differences in categorical variables were compared using *χ* ^2^ or Fisher’s exact test. Logistic regression analysis was used to identify the association between glomerular C3 deposits and 2010 RPS DN classification. A Cox proportional hazard model was used to calculate the hazard ratios (HRs) and 95% confidence intervals (CIs) for variables related to the composite outcome. First, we performed a univariate analysis to estimate the association of baseline variables with the composite outcome. Clinically significant variables and those with a *p*-value <0.1 in the univariate analysis were added individually to the multivariate models. Model 1 included age, sex, and diabetes duration. Model 2 included the variables in model 1 plus diabetic retinopathy, serum albumin, proteinuria, and eGFR. Finally, model 3 was established after adjustment for ACEI/ARB treatment in addition to the variables included in model 2. Adjusted cumulative renal survival curves were plotted after adjusting for the same variables as in the final Cox model. To develop a quantitative tool to estimate the individual post-diagnosis event-free survival probability at 1 and 3 years, a nomogram was constructed based on the combination of DN class and C3 deposits. In addition, time-dependent receiver operating characteristic (ROC) curves were used to predict the prognostic accuracy of markers with respect to the composite outcome. Statistical analyses were performed using SPSS (version 23.0; IBM Corp., Armonk, NY, USA) and R (version 4.0.5; Foundation for Statistical Computing, Vienna, Austria; http://www.R-project.org) software. Differences were considered significant if *p <*0.05.

## Results

### Baseline Characteristics Stratified by Glomerular C3 Deposits

We studied 217 native kidney biopsy specimens from patients with a pathological diagnosis of DN and excluded those with evidence of any other glomerular disease. The clinical and pathological characteristics of the study patients are presented in **Table 1**. The median age at the time of renal biopsy was 53 (46–60) years, and 165 (76%) patients were male. The median eGFR was 47.3 (29.4–67.0) ml/min/1.73 m^2^. The median UPE was 5.1 (2.6–7.5) g/day. A total of 204 patients (94%) had type 2 diabetes. In addition, 174 (80.2%) and 47 (21.7%) of patients had diabetic retinopathy and peripheral neuropathy, respectively.

**Table 1:** Baseline characteristics of diabetic nephropathy stratified by glomerular C3 deposits.

Variables	Total (*n* = 217)	Glomerular C3 deposits			*p*
		C3 negative (*n* = 100)	C3 trace/1+ (*n* = 55)	C3 2+ or higher (*n* = 62)	
Age (years)	53 (46–60)	54 (45–59)	52 (46–59)	53 (46–61)	0.79
Male, *n* (%)	165 (76.0)	76 (76.0)	44 (80.0)	45 (72.6)	0.64
Body mass index (kg/m^2^)	26.5 (23.7–28.4)	26.5 (24.2–28.2)	25.3 (22.6–27.7)	26.8 (23.8–28.4)	0.49
Type 1/type 2 diabetes	13/204	3/97	3/52	7/55	
Duration of diabetes (years)	10 (6–15)	10 (6–15)	10 (6–16)	10 (5–16)	0.74
Hypertension, *n* (%)	182 (83.8)	84 (84.0)	46 (83.6)	52 (83.8)	0.98
MAP (mmHg)	102 (93–110)	102 (93–113)	100 (91–107)	104 (95–110)	0.15
DR, *n* (%)	174 (80.2)	73 (73.0)	47 (87.0)	54 (87.1)	0.034
DPN, *n* (%)	47 (21.7)	21 (21.0)	12 (21.8)	14 (22.6)	0.97
Cardiovascular events, *n* (%)	55 (25.4)	24 (24.0)	12 (21.8)	19 (30.6)	0.5
Hematuria, *n* (%)	69 (31.8)	25 (25.0)	20 (36.4)	24 (38.7)	0.13
Urinary protein excretion (g/day)	5.1 (2.6–7.5)	4.5 (2.5–7.3)	5.4 (2.7–7.9)	5.8 (3.1–7.6)	0.23
HbA1c (%)	7.1 (6.2–8.2)	7.1 (6.2–8.5)	7.0 (6.3–7.9)	7.0 (6.1–7.9)	0.24
Hemoglobin (g/L)	111 (101–131)	117 (103–134)	111 (98–130)	109 (101–125)	0.21
Fasting blood glucose (mmol/L)	7.1 (5.4–8.9)	7.3 (5.6–9.3)	6.7 (5.0–9.1)	7.4 (5.4–8.3)	0.4
eGFR (ml/min/1.73m^2^)	47.3 (29.4–67.0)	48.9 (30.8–72.3)	47.9 (30.2–74.6)	45.9 (26.0–63.0)	0.48
BUN (mmol/L)	8.8 (6.8–11.9)	8.2 (6.4–11.8)	9.2 (7.4–12.1)	9.5 (7.1–11.7)	0.27
Uric acid (μmol/L)	391.5 ± 97.4	388.6 ± 107.1	393.3 ± 86.7	394.5 ± 91.1	0.92
Serum albumin (g/L)	33.6 ± 6.3	34.9 ± 6.5	33.2 ± 6.3	32.1 ± 5.7	0.017
Triglyceride (mmol/L)	1.8 (1.3–2.6)	1.9 (1.3–3.1)	1.8 (1.3–2.1)	1.7 (1.5–2.7)	0.21
Total cholesterol (mmol/L)	5.1 (4.1–6.1)	4.9 (4.0–6.1)	4.9 (4.0–6.0)	5.4 (4.4–6.5)	0.2
HDL-c (mmol/L)	1.1 (0.9–1.2)	1.0 (0.8–1.2)	1.1 (0.9–1.2)	1.1 (0.9–1.3)	0.29
LDL-c (mmol/L)	3.1 (2.4–3.9)	3.0 (2.3–3.7)	3.1 (2.3–3.9)	3.4 (2.6–4.2)	0.13
Serum C3 (mg/dl)	90.8 (77.3–104)	93.5 (82.5–107)	91.2 (76.8–108)	85.6 (74.1–100.3)	0.072
Serum C4 (mg/dl)	25.8 (21.5–30.0)	25.1 (21.4–29.9)	26.1 (21.2–30.6)	25.8 (21.6–29.9)	0.83
Serum C1q (mg/dl)	19.7 (16.2–21.7)	20.1 (16.6–22.8)	18.0 (15.4–21.2)	20.2 (16.3–21.7)	0.17
Using ACEI or ARB, *n* (%)	153 (70.5)	70 (70.0)	40 (72.7)	43 (69.4)	0.91
2010 RPS DN classification					
RPS class (I/IIa/IIb/III/IV)	0/17/67/113/20	0/14/32/47/7	0/3/20/26/6	0/0/15/40/7	0.019
IFTA (0/1/2/3)	0/71/119/27	0/39/52/9	0/17/31/7	0/15/36/11	0.26
Interstitial inflammation (0/1/2)	5/140/72	4/69/27	0/35/20	1/36/25	0.23
Arteriolar hyalinosis (0/1/2)	42/28/147	20/15/65	10/10/35	12/3/47	0.23
Arteriosclerosis (0/1/2)	33/104/80	21/49/30	7/26/22	5/29/28	0.13
Global sclerosis (%)	15.4 (5.1–33.6)	14.3 (2.2–31.8)	12.5 (3.3–33.3)	19.1 (6.8–36.6)	0.41

DR, diabetic retinopathy; DPN, diabetic peripheral neuropathy; MAP, mean artery pressure; HbA1c, glycated hemoglobin A1c; ACEI, angiotensin-converting enzyme inhibitor; ARB, angiotensin II type 1 receptor blocker; DN, diabetic nephropathy; PRS, Renal Pathology Society; BUN, blood urea nitrogen; eGFR, estimated glomerular filtration rate; IFTA, interstitial fibrosis and tubular atrophy.

Direct immunofluorescence microscopy for C3 staining showed completely negative, trace/1+, and ≥2+ staining in 100 (46.1%), 55 (25.3%), and 62 (28.6%) patients, respectively. C3 deposition was observed predominantly in the mesangium (111 out of 117 patients; 94.9%), with a few patients showing deposits in glomerular capillary walls and Bowman’s capsule (6 out of 117; 5.1%). Patients with glomerular C3 deposition had a higher prevalence of diabetic retinopathy, lower serum albumin level, lower serum C3 level, and more severe pathologic phenotype of DN than those without such deposition. No statistical significance was observed in any other clinical and pathological variables among the complement groups.

### Clinical Outcomes Stratified by Glomerular C3 Deposits

**Figure 2:**
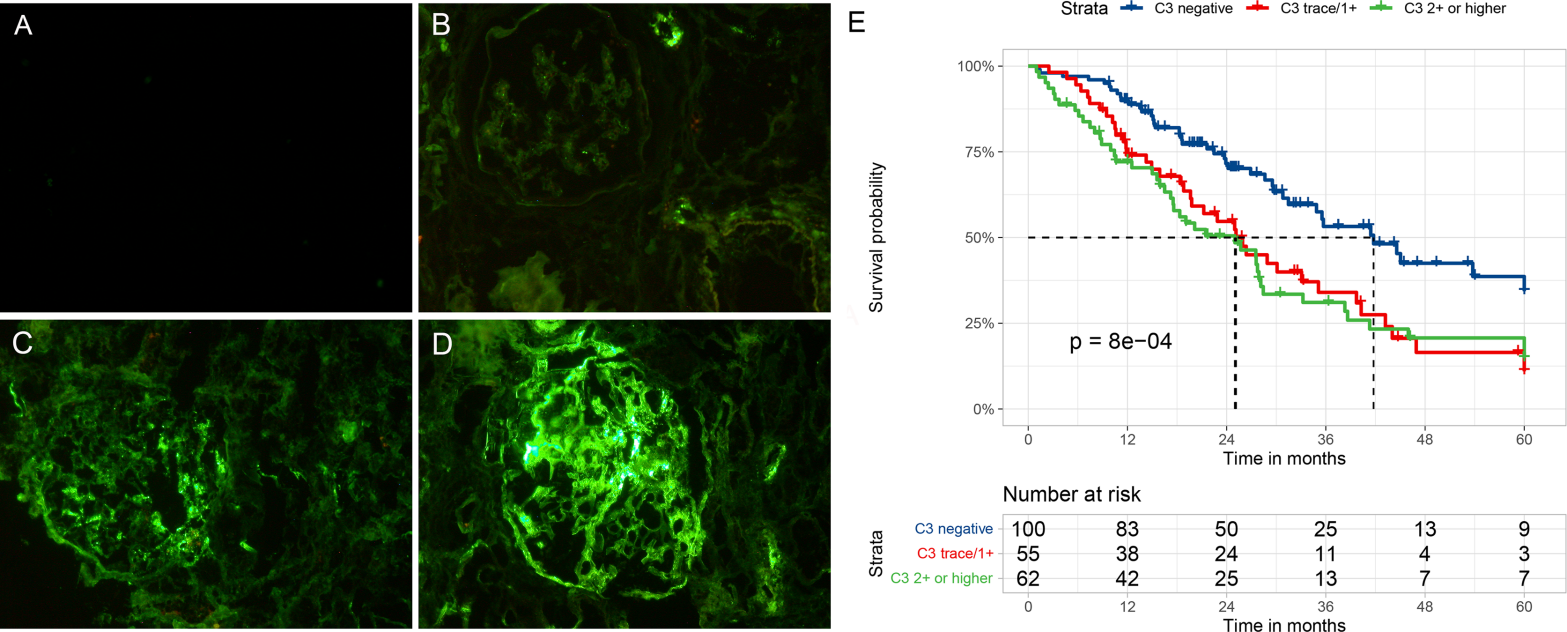
Representative immunofluorescent staining **(A–D)** for C3 in patients with diabetic nephropathy and Kaplan–Meier survival curve **(E)** for a composite of end-stage renal disease, ≥50% decline in initial eGFR, or death according to glomerular C3 deposits. **(A)** No C3 staining (C3–), **(B)** seemingly visible at low magnification but clearly visible at high magnification (C3+), **(C)** clearly visible at both low and high magnification (C3 2+), and **(D)** clearly visible at low magnification but dazzling at high magnification (C3 3+).

**Figures 2A–D** illustrate the varying degrees of C3 staining in glomeruli. **Figure 2E** shows the Kaplan–Meier survival curve for the composite outcome stratified by glomerular C3 deposits. The clinical outcomes during follow-up are presented in **Table 2**. In total, 49 patients were followed up for more than 3 years, and only 20 were followed up for 5 years. During follow-up, the composite outcome occurred in 42 (42%), 37 (67.3%), and 44 (71%) patients with negative, trace/1+, and ≥2+ glomerular C3 stain, respectively. Ten patients died during follow-up: two died before and eight after reaching ESRD. The two patients who died before progression to ESRD had already suffered a ≥50% decline in eGFR. The median follow-up durations for patients with negative, C3 trace/1+, and ≥2+ C3 stain were 24.0 (14.6–37.1), 19.8 (11.5–33.1), and 18.7 (8.8–28.9) months, respectively. As shown in **Figure 2E**, negative C3 deposition was associated with a significantly lower risk of an adverse kidney outcome (*p* < 0.001) compared with other deposition grades. However, the event-free survival probability did not differ between trace/1+ and ≥2+ C3 stain patients (**Figure 2E**; **Supplementary Table S1**). The median event-free survival times for patients with negative, trace/1+, and ≥2+ C3 stain were 41.8 (95% CI, 32.2–51.3), 25.1 (95% CI, 19.1–31.2), and 25.0 (95% CI, 14.8–35.2), respectively. There was no difference in survival time between trace/1+ and ≥2+ C3 stain. Therefore, we used a binary C3 staining intensity classification (negative vs. positive; C3 – and trace/≥1+, respectively) in the following analyses.

**Table 2:** Follow-up data stratified by glomerular C3 deposits.

Outcomes	Total(*n* = 217)	Glomerular C3 deposits		
		C3 negative (*n* = 100)	C3 trace/1+ (*n* = 55)	C3 2+ or higher (*n* = 62)
Composite outcome, *n* (%)	123 (56.7)	42 (42.0)	37 (67.3)	44 (71.0)
≥50% decline in eGFR, *n* (%)	21 (9.7)	11 (11.0)	5 (9.1)	5 (8.1)
End-stage renal disease, *n* (%)	108 (49.8)	38 (38.0)	32 (58.2)	38 (61.3)
Death, *n* (%)	10 (4.6)	4 (4.0)	1 (1.8)	5 (8.1)
Follow-up (months)	21.6 (12.0–33.6)	24.0 (14.6–37.1)	19.8 (11.5–33.1)	18.7 (8.8–28.9)

eGFR, estimated glomerular filtration rate.

### Association of C3 Deposits With the RPS DN Classification

Next, we examined the association between C3 deposits and RPS classification using multivariate logistic regression analysis. We found that C3 deposits had a significant correlation with glomerular class [odds ratio (OR), 1.59 (95% CI, 1.08–2.36); *p* = 0.02]. As illustrated in **Supplementary Figure S1**, the incidence of C3 deposits in class IIa patients (17.6%) was significantly lower than in class IIb (52.2%), III (58.4%), and IV (65%) patients (*p* < 0.05). In classes IIb–IV patients, C3 deposition showed a gradually increasing trend, albeit in a non-significant manner. However, the ORs for C3 deposits in the presence of IFTA (scores 2–3), interstitial inflammation (score 2), arteriolar hyalinosis (score 2), and arteriosclerosis (score 2) were 1.19 (95% CI, 0.63–2.26), 1.48 (95% CI, 0.79–2.78), 0.79 (95% CI, 0.4–1.58), and 1.52 (95% CI, 0.78–2.99), respectively (**Table 3**). This suggests that the degree of interstitial and vascular involvement in DN was not associated with glomerular C3 deposits in DN patients.

**Table 3:** Multivariable-adjusted logistic regression analysis for glomerular C3 deposits according to the 2010 RPS DN classification.

RPS DN classification	Odds ratio (95% CI)	*p*
RPS class (IIa/IIb/III/IV)	1.59 (1.08–2.36)	0.02
IFTA (2–3)^a^	1.19 (0.63–2.26)	0.59
Interstitial inflammation (2)^a^	1.48 (0.79–2.78)	0.22
Arteriolar hyalinosis (2)^a^	0.79 (0.40–1.58)	0.51
Arteriosclerosis (2)^a^	1.52 (0.78–2.99)	0.22

a For analysis, scores of 2 and 3 were collapsed together as 2 for IFTA, and scores of 0 and 1 were collapsed together as 1 for interstitial inflammation, arteriolar hyalinosis, and arteriosclerosis.

RPS, Renal Pathology Society; DN, diabetic nephropathy; IFTA, interstitial fibrosis and tubular atrophy.

### Correlations of C3 Deposits and RPS Class With the Composite Outcome in DN

During a median follow-up of 22 months, 123 (56.7%) patients reached the composite outcome. The composite outcome occurred more frequently in classes IIb (52.2%), III (60.2%), and IV (85%) patients compared with class IIa (17.6%) patients. Similarly, patients with glomerular C3 deposition (69.2%) had a higher incidence of the composite outcome compared with those without complement deposition (42%). In the multivariate Cox model, after adjusting for age, sex, diabetes duration, diabetic retinopathy, serum albumin, proteinuria, eGFR, and ACEI/ARB treatment, the HRs for classes IIb, III, and IV (IIa as the reference) and glomerular C3 deposition were 3.29 (95% CI, 0.99–10.98; *p* = 0.05), 3.96 (95% CI, 1.23–12.79; *p* = 0.021), 5.37 (95% CI, 1.52–18.90; *p* = 0.009), and 1.82 (95% CI, 1.23–2.68; *p* = 0.002), respectively (**Table 4**).

**Table 4:** Hazard ratios for a composite of ESRD or 50% decline in initial eGFR stratified by glomerular C3 deposits or RPS class.

	Events/total (%)	Unadjusted		Model 1		Model 2		Model 3	
		HR (95% CI)	*p*	HR (95% CI)	*p*	HR (95% CI)	*p*	HR (95% CI)	*p*
RPS class									
Class IIb	35/67 (52.2)	4.66 (1.43–15.18)	0.011	4.41 (1.35–14.39)	0.014	3.02 (0.91–9.99)	0.07	3.29 (0.99–10.98)	0.05
Class III	68/113 (60.2)	5.55 (1.75–17.68)	0.004	5.29 (1.66–16.89)	0.005	3.78 (1.17–12.17)	0.026	3.96 (1.23–12.79)	0.021
Class IV	17/20 (85.0)	12.07 (3.53–41.29)	<0.001	11.32 (3.29–38.98)	<0.001	5.16 (1.47–18.11)	0.01	5.37 (1.52–18.90)	0.009
C3 deposition	81/117 (69.2)	1.97 (1.36–2.86)	<0.001	2.06 (1.40–3.02)	<0.001	1.79 (1.22–2.64)	0.003	1.82 (1.23–2.68)	0.002

Model 1: adjusted for age, sex, and duration of diabetes. Model 2: model 1 + diabetic retinopathy, serum albumin, proteinuria, and eGFR. Model 3: model 2 + ACEI/ARB treatment.

HR, hazard ratio; CI, confidence interval; ESRD, end-stage renal disease; eGFR, estimated glomerular filtration rate; RPS, Renal Pathology Society.

### Combined Effects of C3 Deposits and RPS Class on the Composite Outcome in DN

**Figure 3:**
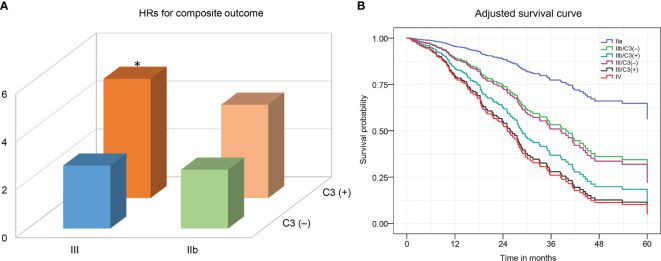
The hazard ratios **(A)** and adjusted-cumulative survival curve **(B)** for a composite of ESRD, ≥50% decline in initial eGFR, or death according to the combination of C3 deposits and the 2010 RPS DN classification. The Cox proportional hazards model was adjusted for age, sex, duration of diabetes, diabetic retinopathy, serum albumin, proteinuria, eGFR, and ACEI/ARB treatment. ESRD, end-stage renal disease; eGFR, estimated glomerular filtration rate. RPS, Renal Pathology Society; DN, diabetic nephropathy. *p <0.05 vs. class III/C3(–).

We evaluated whether the combination of glomerular C3 deposition and RPS class could have a greater adverse effect on deteriorating kidney function in DN patients. Because there were too few patients categorized as classes IIa (*n* = 17) and IV (*n* = 20), meaningful subgroup analyses could not be performed, and only classes IIb (*n* = 67) and III (*n* = 113) were further categorized into subgroups based on the presence of C3 deposition. The composite outcome occurred more frequently in patients with C3 than those without deposition (class IIb, 62.9% vs. 40.6%; class III, 72.7% vs. 42.6%). The results of the univariate and multivariate Cox proportional hazards models are displayed in **Supplementary Table S2**. Classes IIb and III patients with C3 deposition had a higher risk of adverse kidney outcome than those with absent C3 deposition (**Figure 3**; **Supplementary Table S2**). The HRs were 2.46 (95% CI, 0.68–8.89; *p* = 0.17) for class IIb only, 3.9 (95% CI, 1.14–13.17; *p* = 0.03) for class IIb and C3 deposition, 2.63 (95% CI, 0.77–9.0) for class III only, and 4.98 (95% CI, 1.53–16.23; *p* = 0.008) for class III and C3 deposition compared with class IIa (88% of patients with class IIa had no C3 deposition).

**Figure 4:**
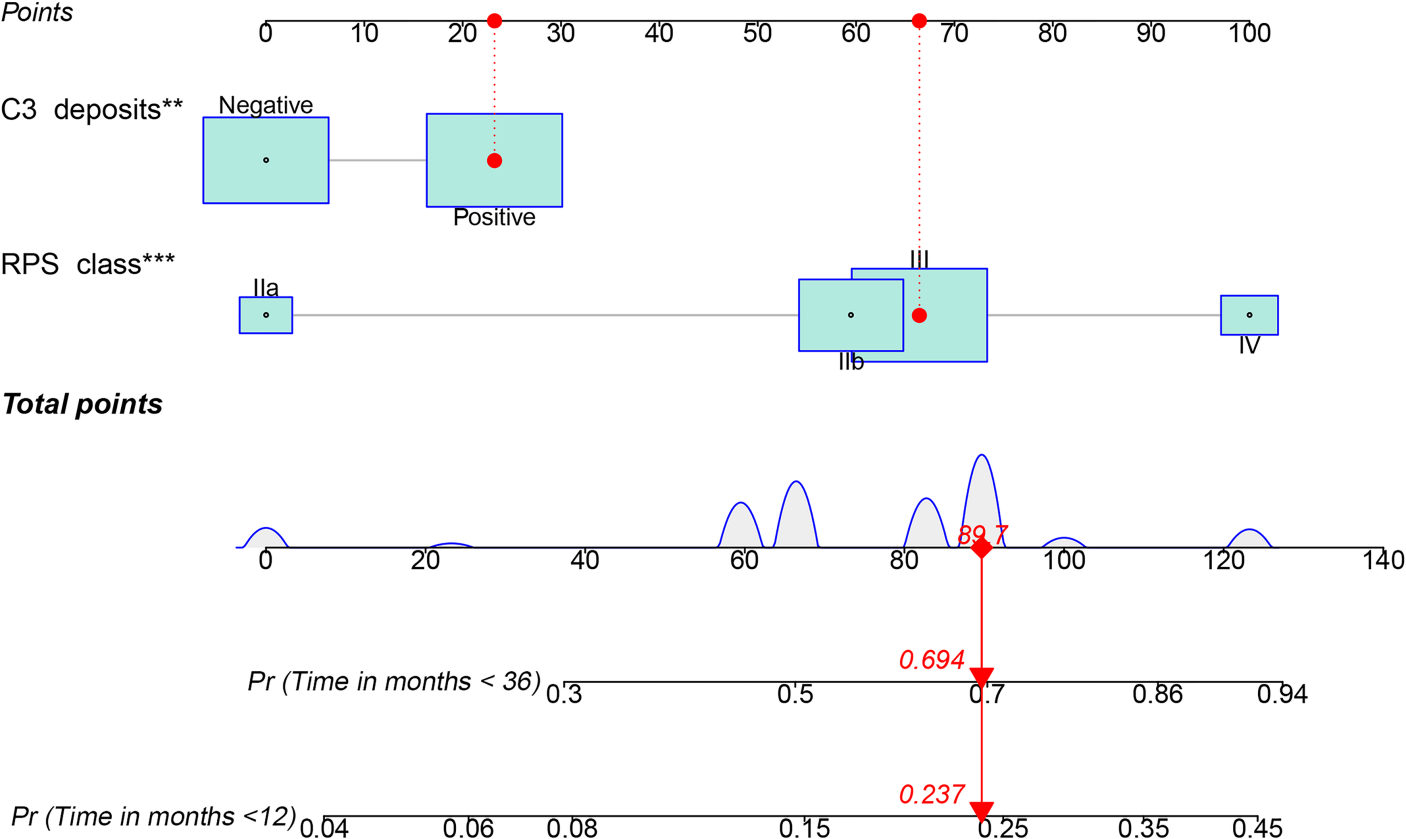
Nomogram predicting the occurrence of a composite of end-stage renal disease, ≥50% decline in initial estimated glomerular filtration rate, or death according to the combination of C3 deposits and RPS class. PRS, Renal Pathology Society; ^**^ *p* < 0.01; ^***^ *p* < 0.001. C3 positive, 23.2 points; IIb, 59.5 points; III, 66.4 points; IV, 100 points.

A nomogram was developed for the composite outcome based on C3 deposits and RPS class (**Figure 4**), which provides a more intuitive assessment of survival for individual DN patients—for example, a class III DN patient with no C3 deposits would have a total score of 66.4, which corresponds to 15 and 50% probabilities of adverse kidney outcome within 1 and 3 years, respectively. However, if this patient had coincident C3 deposits, the probabilites of adverse kidney outcome are 24 and 69% within 1 and 3 years, respectively.

### Prognostic Accuracy of RPS Class, C3 deposits, and the Combination of RPS Class and C3 Deposits

**Figure 5:**
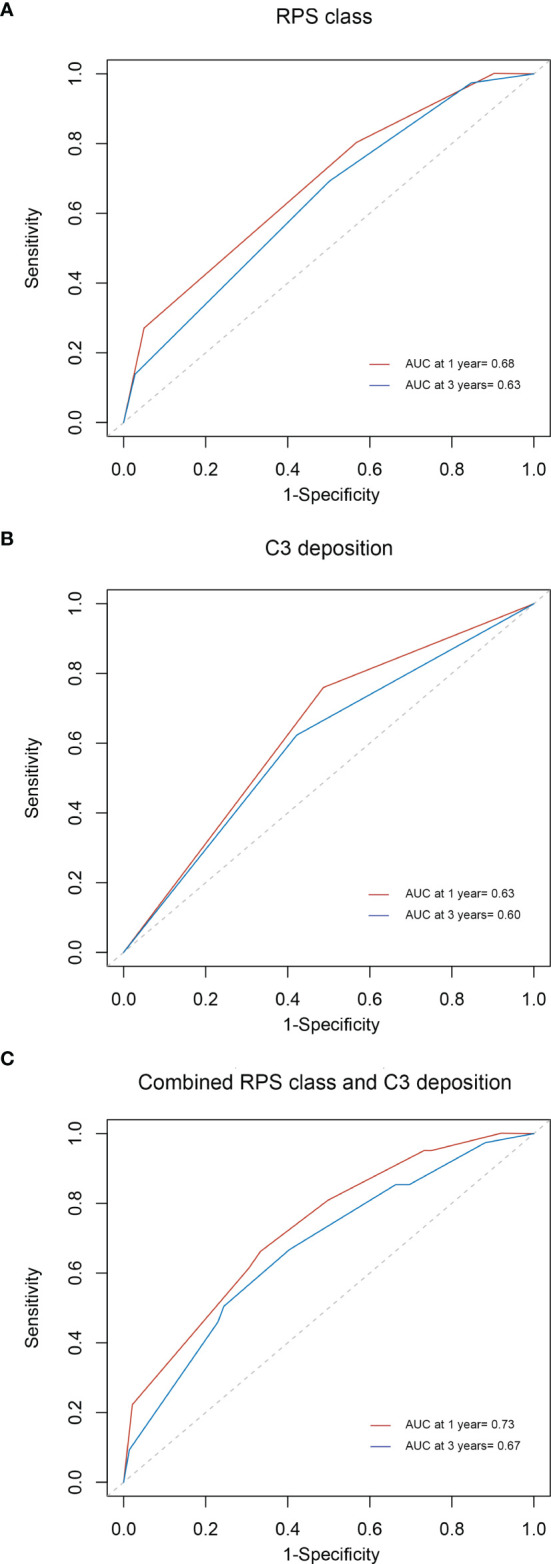
Time-dependent ROC curves for the estimation of prognostic accuracy with RPS class **(A)**, C3 deposition **(B)**, or the combination of C3 deposition and RPS class **(C)** in diabetic nephropathy. ROC, receiver operating characteristic; RPS, Renal Pathology Society.

To study the prognostic accuracy of a single biomarker, measured at baseline, on the onset of a disease condition when the disease onset may occur at different times during follow-up, efficient estimators of time-dependent sensitivity and specificity as well as area under the ROC curve (AUC) were implemented in R packages. As shown in **Figure 5A**, the AUCs of RPS class for predicting the composite outcome at 1 and 3 years were 0.68 and 0.63, respectively. **Figures 5B, C** display the AUCs of C3 deposits (0.63 and 0.60 at 1 and 3 years, respectively) and of RPS class and C3 deposits combined (0.73 and 0.67 at 1 and 3 years, respectively). The AUC values of RPS class and C3 deposits combined were higher than those of RPS class and C3 deposits alone.

In addition to C3 deposition and RPS class, other parameters, including UPE, initial eGFR, and ACEI/ARB therapy, can also predict disease progression. A time-dependent ROC curve integrating the independent predictors for renal survival is displayed in **Supplementary Figure S2**. The AUCs of the aforementioned combined predictors of disease progression at 1 and 3 years were 0.85 and 0.86, respectively.

### Association of C3 Deposits With IgM and C1q Deposits

**Figure 6:**
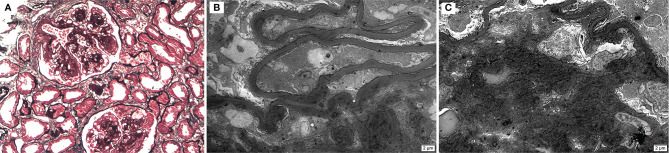
Diabetic nephropathy with Kimmelstiel–Wilson nodule **(A)** (light microscopy, ×200), and prominent thickening of glomerular basement membranes with expanded mesangium and a small amount of mesangial electron-dense deposits **(B–C)** (electron microscopy; ×5,000).

Because of the frequent deposition of C1q and IgM in the expanded mesangial matrix and their relatively high molecular weights, they are often considered to be uncharacteristically trapped. Therefore, we explored whether there was a relationship between C3 and C1q and IgM. Similar to C3 deposition, the deposition of C1q and IgM was considered positive if immunofluorescence intensity was detected at the trace or ≥1+ level in the glomerular compartment. IgM and C1q staining were positive in 148 (68.2%; 146 in the mesangium and 2 in the glomerular capillary walls) and 44 (20.3%; all located in mesangium) patients, respectively. Of the 117 patients with C3 deposits, 89 (76%) had co-deposits of IgM and 39 (33%) had co-deposits of C1q. Of the 100 patients without C3 deposits, 59 (59%) had IgM deposits and 5 (5%) had C1q deposits (**Supplementary Table S3**). The correlations of C3 deposition with these two markers were examined using multivariate logistic regression analysis. The OR for C3 deposition in the presence of IgM and C1q was 1.59 (95% CI, 0.86–2.95; *p* = 0.13) and 8.4 (95% CI, 3.12–22.61; *p* < 0.001), respectively (**Table 5**). These findings suggest no significant correlation between C3 and IgM deposition but a significant correlation between C3 and C1q, which argues for a specific C3 deposition in the mesangium and involvement of the classic complement pathway. **Figure 6** displays several light and electron microscopy pictures of one case with C3 deposition under immunofluorescence microscopy (C3 2+; C1q 1+; IgM ±). It should be noted, however, that transmission electron microscopy does not distinguish the composition of the dense substances.

**Table 5:** Logistic regression analysis for glomerular C3 deposits according to the data of C1q and IgM deposits.

Staining findings of immunofluorescence	Odds ratio (95% CI)	*p*
C1q deposits	8.40 (3.12–22.61)	<0.001
IgM deposits	1.59 (0.86–2.95)	0.13

Either C1q or IgM deposition was taken as positive if an immunofluorescence intensity of them was detected at a trace level or ≥1+ in the glomerular compartment.

### Association of C3 Deposits With Proteinuria

**Figure 7:**
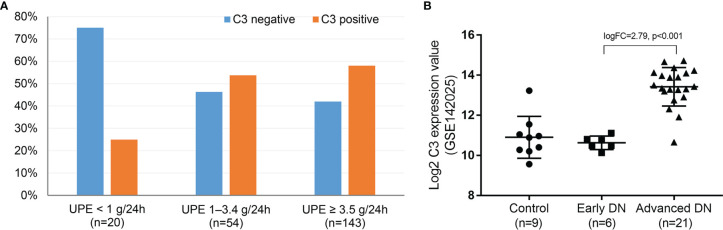
Direct immunofluorescence for the proportion of C3 deposits at different proteinuria levels in DN **(A)** and RNA sequencing analysis of C3 from the whole-kidney tissues of patients with early and advanced DN compared with nephrectomy sample tissues from those without diabetes **(B)**. DN, diabetic nephropathy. Early DN was defined as urinary albumin-to-creatinine ratio (UACR) between 30 and 300 mg/g and estimated glomerular filtration rate (eGFR) >90 ml/min/1.73 m^2^. Advanced DN was defined as UACR >300 mg/g and eGFR <90 ml/min/1.73 m^2^. The control human kidney samples were obtained from the unaffected portion of tumor nephrectomies.

The relationship between C3 expression and UPE level was explored. As shown in **Figure 7A**, direct immunofluorescence staining indicated that the proportion of C3 deposits in DN patients with UPE <1 g/24 h (25%) was significantly lower than that in patients with UPE of 1–3.5 g/24 h (53.7%) or >3.5 g/24 h (58%) (*p* < 0.05). In addition, we downloaded the kidney transcription profiles of DN patients from the Gene Expression Omnibus database (http://www.ncbi.nlm.nih.gov/geo). GSE142025 enabled the RNA sequencing analysis of kidney tissues from DN patients with micro- [urinary albumin-to-creatinine ratio (UACR) of 30–300 mg/g] and macro-albuminuria (UACR >300 mg/g) compared with unaffected tissues obtained from the tumor nephrectomies of patients without diabetes. C3 was significantly overexpressed in the kidney tissue of patients with advanced DN (macroalbuminuria and eGFR <90 ml/min/1.73 m^2^) compared with those with early DN (microalbuminuria and eGFR >90 ml/min/1.73 m^2^) and healthy controls (*p* < 0.001; **Figure 7B**). These findings suggest that complement activation may be primarily involved in DN progression rather than DN onset.

## Discussion

This is the first published series to investigate the correlation between C3 deposits in the biopsy specimens of DN patients and the RPS DN classification as well as the combined deleterious effects thereof on kidney function. This study had several important findings: C3 deposition in DN kidneys is common, early stage (*e*.*g*., IIa) DN has a lower incidence of complement deposition compared with late-stage DN, and patients with C3 deposition and RPS class have a poor prognosis. Our findings highlight the importance of combining complement deposition with DN class for the clinical prediction of the likelihood of developing diabetic ESRD. Additionally, we did not find a correlation between glomerular complement deposits and the lesion scores of the tubulointerstitial and vascular compartments.

In clinical practice, DN patients generally do not undergo biopsy unless other causes of kidney disease that require specific therapy are suspected. Therefore, data regarding the downstream phenotype of DN are limited. Herein we summarized the biopsy data obtained over nearly a decade and found that patients with diabetes underwent kidney biopsy at a median eGFR of 47 ml/min/1.73 m^2^ and proteinuria of 5.1 g/day. This suggests that kidney biopsies tend to be performed in the late stages of diabetes. As a result, the number of classes I and IIa patient was very limited, and interstitial fibrosis and inflammation were also largely involved.

The RPS classification system combines type 1 and type 2 DN and can distinguish between lesions varying in severity. This system is widely used in clinical practice (7, 9, 15, 16). The pathogenesis of DN lesions is complex and involves various pathways. Using the RPS classification to evaluate the biopsies of DN patients may unravel the complex pathways that ultimately lead to glomerulosclerosis in DN. Our previous study showed that RPS class is predictive of time to ESRD (6), consistent with the findings of many previous studies and supporting the use of PRS class as a prognostic marker (15, 17, 18). However, some studies reported that when DN classes I and IIa were removed from the analyses, RPS class was not an independent predictor of diabetic ESRD (19). In fact, for this 120-month study, the median follow-up duration was only 18 months, indicating excessively censored data and the need for further validation of the results.

Interestingly, growing evidence suggests a pathogenic role of the complement system in the development and clinical outcomes of DN (6–8), which, in turn, suggests that complement activation might not only identify patients at risk of such complications but may also be a therapeutic target. Given that complement C3 is key molecule in complement activation in the three pathways and forms the basis of a positive feedback loop that involves C3b, we studied whether glomerular C3 deposits are linked to morphological features stratified by RPS classification. This study is significant because it is the first to report that complement deposition affects DN prognosis through synergistic effects with pathological phenotypes of DN rather than through complement deposition or individual lesions in the RPS class alone.

In this study, we observed that complement deposition was not associated with lesion severity in the tubulointerstitial and vascular compartments but was associated with RPS class. One possible explanation for this is that complement deposition was defined on the basis of glomerular deposits instead of deposition in the tubulointerstitium and vasculature. The vast majority of C3 deposition occurred in the mesangial region. The mechanism underlying the synergistic effects of complement deposition and RPS class has been reported in previous experimental studies. C3aR is expressed in podocytes, and complement activation leads to an increased expression of C3a. C3a/C3aR signaling on podocytes promotes the production of IL-1β, thereby inducing podocyte cytoskeleton rearrangement and loss in an autocrine manner, which, in turn, lead to diabetic glomerulosclerosis (20). Additionally, complement factor B mediates podocyte injury in DN *via* the mTOR complex 1 signaling pathway (21). Finally, C5b-9, the end-product of complement activation, inserts into cell membranes (*e*.*g*., podocytes and mesangial cells) and causes cellular injury and tissue inflammation, which, in turn, result in sclerosis and fibrosis in DN (22–24). However, DN is not primarily a disease of podocytes. C3 negativity in 100 of the 217 cases in this study clearly argues against a pathogenetic role of the complement system in DN. It is possible that patients with a low threshold for complement activation have a tendency (probably due to genetic factors) for worsening glomerular diseases, including DN.

The deposition of a single immunofluorescence marker in a markedly expanded mesangial matrix does not necessarily suggest local complement activation. Although the present study showed the prognostic significance of C3 deposition, independent of RPS class, it is possible that trapping correlates with the amount of extracellular matrix, which likely varies significantly between cases within the same RPS class. To investigate this, we evaluate the immunofluorescence data for C1q and IgM since these markers are frequently deposited in the expanded mesangial matrix, have relatively high molecular weights, and are often considered trapped. In this study, most patients with C1q deposits had concurrent C3 deposits (88.6%). However, among patients with C3 deposits, only 33.3% had co-deposits of C1q. Moreover, no significant correlation was observed between C3 and IgM deposition, although there was a correlation between C3 and C1q suggesting specific C3 deposition in the mesangium and classic complement activation in DN. Since C3 deposition indicates the activation of a common complement pathway, pathways other than the classic pathway may also be activated. Different pathways may have varying degrees of involvement in the development of DN. Indeed previous research has shown that the activation of the alternative and lectin pathways may also be involved in the pathogenesis of DN (25–27). Given the role of the complement system in the pathogenesis of DN, inhibition of the specific components of the complement system may be an effective therapeutic strategy for DN (28).

Our study has several limitations. First, as it was an observational study, causality was difficult to infer. Moreover, it included a relatively small sample drawn from a single institution. Nevertheless, this is still one of the largest published series of biopsy patients to date. Second, our study samples were mainly from the clinical biopsies of patients with late-stage disease (predominantly type 2 DN). Therefore, a large multicenter study including a broad spectrum of disease severity is required. Despite this, we demonstrated that the complement system could affect the downstream phenotype of DN. Third, C3 deposition was observed and investigated on the basis of the glomerular compartment, without considering tubular and vascular complement deposits, which might also mediate the kidney injury of DN. Finally, no specific staining was performed for the other complement components, including C3a, C5a, and C5b-9. Future studies should explore these issues in detail, and the relationship between the presence of C3 and pathological changes can also be investigated under immunoelectron microscopy.

## Conclusion

In summary, we showed that glomerular C3 deposits are associated with worsening kidney function and correlate with the pathogenic phenotype of DN. The combination of C3 deposits and RPS class allows for a more accurate prediction of the time to diabetic ESRD. Therefore, the use of C3 deposition enhances the clinical utility of the RPS classification for DN.

## Data Availability Statement

The original contributions presented in the study are included in the article/**Supplementary Material**. Further inquiries can be directed to the corresponding author.

## Ethics Statement

The studies involving human participants were reviewed and approved by the Institutional Ethics Committee of China-Japan Friendship Hospital. The patients/participants provided their written informed consent to participate in this study.

## Author Contributions

SJ contributed to the conception and design of the study and wrote the first draft of the manuscript. DD and YJ collected the clinical data. GZ and HG were responsible for histopathological preparation, staining, and reading of results. WL revised the final version and was the guarantor of this work. All authors contributed to the article and approved the submitted version.

## Funding

This work was supported by grants from the Beijing Municipal Natural Science Foundation (7202179), the Science and Technology Project of Beijing (D171100002817003), and National Key Clinical Specialty Capacity Building Project (2019-542).

## Conflict of Interest

The authors declare that the research was conducted in the absence of any commercial or financial relationships that could be construed as a potential conflict of interest.

## Publisher’s Note

All claims expressed in this article are solely those of the authors and do not necessarily represent those of their affiliated organizations, or those of the publisher, the editors and the reviewers. Any product that may be evaluated in this article, or claim that may be made by its manufacturer, is not guaranteed or endorsed by the publisher.
