# Supplementary material for: Complement Deposition Predicts Worsening Kidney Function and Underlines the Clinical Significance of the 2010 Renal Pathology Society Classification of Diabetic Nephropathy
Source: Front Immunol. 2022 May 27;13:868127. doi: 10.3389/fimmu.2022.868127 (PMC9196586; doi:10.3389/fimmu.2022.868127)
Supplement: Supplementary file 1 [file DataSheet_1.docx]

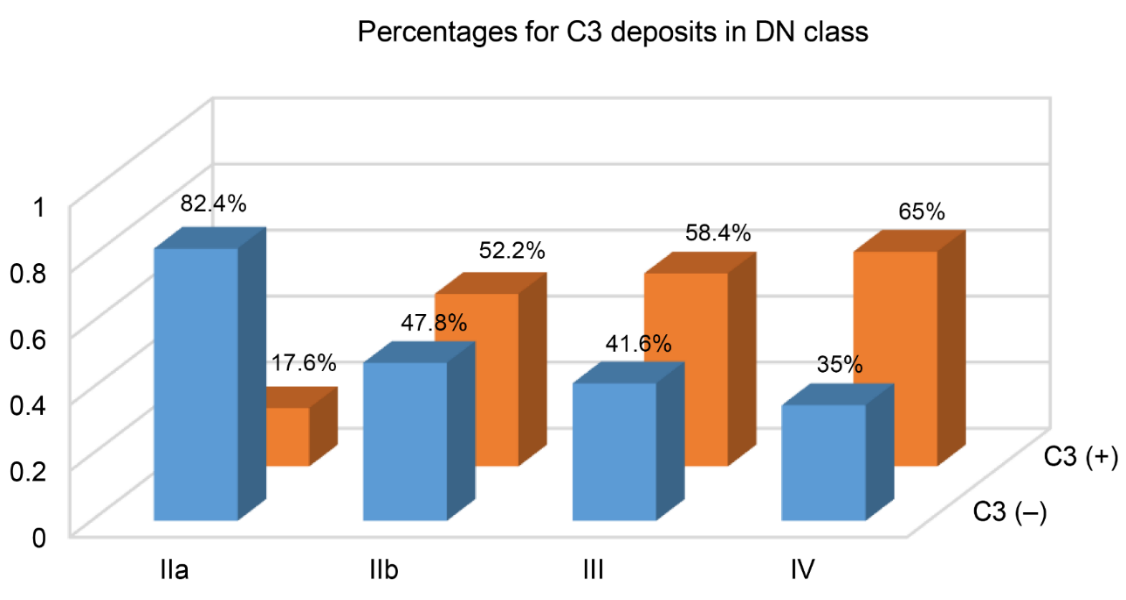


**Supplementary Figure S1.** Percentages of C3 deposits according to RPS class. RPS, Renal Pathology Society.


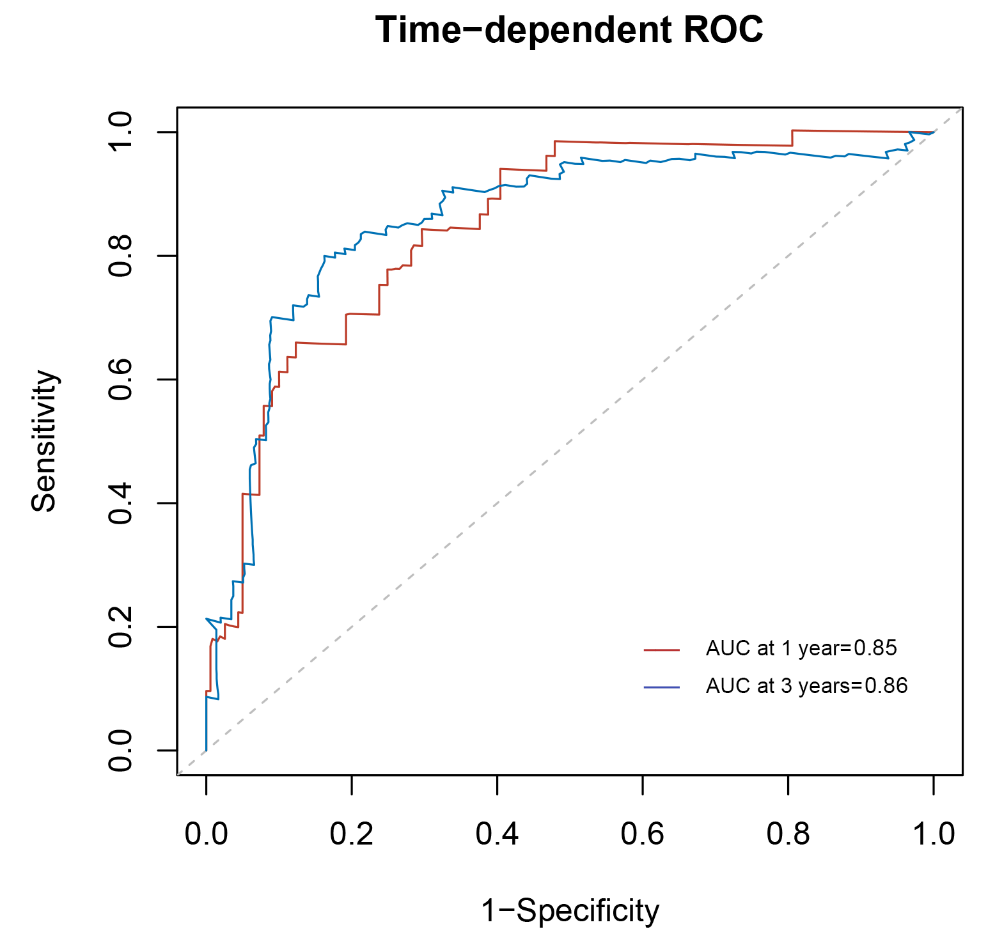


**Supplementary Figure S2.** Time-dependent ROC curves for the estimation of prognostic accuracy with the combination of urinary protein excretion, estimated glomerular filtration rate, ACEI/ARB therapy, C3 deposition and RPS class in diabetic nephropathy. ROC, receiver operating characteristic; RPS, Renal Pathology Society. ACEI, angiotensin-converting enzyme inhibitor; ARB, angiotensin II type 1 receptor blocker.

**Supplementary Table S1.** The hazard ratios for a composite of ESRD, a ≥50% decline in eGFR or death according to C3 deposits

|  | **Trace/1+ versus no C3 deposits** | | |  | **2+ or higher versus no C3 deposits** | | |  | **Trace/1+ versus ≥2+** | | |
| --- | --- | --- | --- | --- | --- | --- | --- | --- | --- | --- | --- |
|  | **HR** | **95% CI** | **p** |  | **HR** | **95% CI** | **p** |  | **HR** | **95% CI** | **p** |
| Unadjusted | 1.92 | 1.23–2.98 | 0.004 |  | 2.04 | 1.33–3.11 | 0.001 |  | 1.09 | 0.70–1.69 | 0.69 |
| Model 1 | 2.05 | 1.29–3.26 | 0.002 |  | 2.06 | 1.34–3.18 | 0.001 |  | 1.07 | 0.68–1.67 | 0.78 |
| Model 2 | 2.03 | 1.28–3.23 | 0.003 |  | 1.76 | 1.12–2.77 | 0.014 |  | 0.97 | 0.62–1.54 | 0.90 |
| Model 3 | 2.07 | 1.30–3.29 | 0.002 |  | 1.86 | 1.18–2.94 | 0.008 |  | 0.99 | 0.62–1.57 | 0.96 |
| Model 1: adjusted for age, sex, and duration of diabetes.  Model 2: Model 1 + diabetic retinopathy, serum albumin, proteinuria and eGFR.  Model 3: Model 2 + ACEI/ARB treatment  eGFR: estimated glomerular filtration rate; ESRD, end-stage renal disease; hazard ratio; CI: confidence interval. | | | | | | | | | | | |

**Supplementary Table S2**. HRs for a composite of ESRD or 50% decline in eGFR according to the combination of C3 deposits and RPS class

|  | **Events/total (%)** | **Unadjusted** | |  | **Model 1** | |  | **Model 2** | |  | **Model 3** | |
| --- | --- | --- | --- | --- | --- | --- | --- | --- | --- | --- | --- | --- |
|  |  | **HR (95%CI)** | **p** |  | **HR (95%CI)** | **p** |  | **HR (95%CI)** | **p** |  | **HR (95%CI)** | **p** |
| Class IIa | 3/17 (17.6) | Reference | | | | | | | | | | |
| Class IIb/C3 (–) | 13/32 (40.6) | 3.66 (1.04–12.85) | 0.043 |  | 3.36 (0.95–11.86) | 0.06 |  | 2.25 (0.63–8.11) | 0.21 |  | 2.46 (0.68–8.89) | 0.17 |
| Class IIb/C3 (+) | 22/35 (62.9) | 5.58 (1.67–18.66) | 0.005 |  | 5.27 (1.57–17.68) | 0.007 |  | 3.57 (1.05–12.11) | 0.041 |  | 3.90 (1.14–13.17) | 0.03 |
| Class III/C3 (–) | 20/47 (42.6) | 3.57 (1.06–12.03) | 0.04 |  | 3.19 (0.94–10.83) | 0.063 |  | 2.57 (0.75–8.80) | 0.13 |  | 2.63 (0.77–9.0) | 0.12 |
| Class III/C3 (+) | 48/66 (72.7) | 7.24 (2.25–23.28) | 0.001 |  | 7.09 (2.20–22.82) | 0.001 |  | 4.71 (1.44–15.35) | 0.01 |  | 4.98 (1.53–16.23) | 0.008 |
| Class IV | 17/20 (85.0) | 12.09 (3.54–41.36) | <0.001 |  | 11.16 (3.24–38.41) | <0.001 |  | 5.07 (1.44–17.80) | 0.011 |  | 5.25 (1.49–18.47) | 0.01 |
| Model 1: adjusted for age, sex, and duration of diabetes.  Model 2: Model 1 + diabetic retinopathy, serum albumin, proteinuria and eGFR.  Model 3: Model 2 + ACEI/ARB treatment  HR, hazard ratio; CI, confidence interval; ESRD, end-stage renal disease; eGFR, estimated glomerular filtration rate; RPS, Renal Pathology Society. | | | | | | | | | | | | |

**Supplementary Table S3.** Percentages of C3 deposits according to IgM or C1q deposits.

| Staining findings of immunofluorescence | | C3 deposits | |
| --- | --- | --- | --- |
|  |  | Negative, n (%) | Positive, n (%) |
| IgM deposits | Negative | 41 (59.4) | 28 (40.6) |
|  | Positive | 59 (39.9) | 89 (60.1) |
| C1q deposits | Negative | 95 (54.9) | 78 (45.1) |
|  | Positive | 5 (11.4) | 39 (88.6) |
